# mtUPR Modulation as a Therapeutic Target for Primary and Secondary Mitochondrial Diseases

**DOI:** 10.3390/ijms24021482

**Published:** 2023-01-12

**Authors:** Paula Cilleros-Holgado, David Gómez-Fernández, Rocío Piñero-Pérez, Diana Reche-López, Mónica Álvarez-Córdoba, Manuel Munuera-Cabeza, Marta Talaverón-Rey, Suleva Povea-Cabello, Alejandra Suárez-Carrillo, Ana Romero-González, Juan Miguel Suárez-Rivero, Jose Manuel Romero-Domínguez, Jose Antonio Sánchez-Alcázar

**Affiliations:** Centro Andaluz de Biología del Desarrollo (CABD-CSIC-Universidad Pablo de Olavide), 41013 Sevilla, Spain

**Keywords:** mitochondria, proteostasis, mitochondrial unfolded protein response, mitochondrial biogenesis, therapeutic target, neurodegenerative diseases, mitochondrial diseases, ageing, metabolic diseases, cardiovascular diseases, cancer

## Abstract

Mitochondrial dysfunction is a key pathological event in many diseases. Its role in energy production, calcium homeostasis, apoptosis regulation, and reactive oxygen species (ROS) balance render mitochondria essential for cell survival and fitness. However, there are no effective treatments for most primary and secondary mitochondrial diseases to this day. Therefore, new therapeutic approaches, such as the modulation of the mitochondrial unfolded protein response (mtUPR), are being explored. mtUPRs englobe several compensatory processes related to proteostasis and antioxidant system mechanisms. mtUPR activation, through an overcompensation for mild intracellular stress, promotes cell homeostasis and improves lifespan and disease alterations in biological models of mitochondrial dysfunction in age-related diseases, cardiopathies, metabolic disorders, and primary mitochondrial diseases. Although mtUPR activation is a promising therapeutic option for many pathological conditions, its activation could promote tumor progression in cancer patients, and its overactivation could lead to non-desired side effects, such as the increased heteroplasmy of mitochondrial DNA mutations. In this review, we present the most recent data about mtUPR modulation as a therapeutic approach, its role in diseases, and its potential negative consequences in specific pathological situations.

## 1. Introduction

Mitochondria are considered the powerhouses of eukaryotic cells by coupling metabolite oxidation through the tricarboxylic acid (TCA) cycle to adenosine triphosphate (ATP) production by oxidative phosphorylation (OXPHOS) [1]. In addition, many other cellular processes are carried out in mitochondria, such as cell death and survival signaling [2], calcium homeostasis [3], cell differentiation [4], redox regulation [5], or the synthesis of cofactors, such as heme groups or iron-sulfur centers, among others [6,7]. These organelles also host various metabolic pathways, such as the urea cycle, β-oxidation of fatty acids, lipid synthesis, and amino acid or nucleotide metabolism [8,9,10]. Mitochondria are semi-autonomous organelles with their own genome, called mitochondrial DNA (mtDNA). Circular mtDNA molecules are located in the mitochondrial matrix associated with the mitochondrial inner membrane and code for 13 proteins involved in the mitochondrial electron transport chain (mtETC), 22 tRNAs, and 2 rRNAs which are necessary for the translation of mtDNA [11]. However, the mitochondrial proteome is composed of approximately 1200 proteins mostly encoded by the nuclear genome [12,13]. Nuclear-encoded mitochondrial proteins are synthesized on cytosolic ribosomes and imported into the mitochondria via the translocase inner membrane (TIM) and translocase outer membrane (TOM) systems [14]. Therefore, both mitochondrial and nuclear genomes, as well as the communication between them, are necessary for the proper function of the mitochondrion. 

The disruption of mitochondrial function is usually caused by the excessive production of reactive oxygen species (ROS), the uncoupling of the mtETC, or the expression of aberrant or mutated proteins encoded by mitochondrial or nuclear DNA (nDNA). In addition, mtDNA is more susceptible to mutations due to its proximity to the site of ROS generation and the absence of histone protection [15,16,17]. These perturbations are implicated in primary mitochondrial diseases, which are characterized by mutations that affect the nDNA or mtDNA, as well as various age-related diseases, metabolic disorders, heart pathologies, and cancer, which are referred to as secondary mitochondrial diseases [18,19,20,21]. 

Mitochondrial homeostasis and proteostasis (the homeostasis of the proteome) are essential for the maintenance of mitochondrial function. To this end, mitochondria have renewal mechanisms, such as mitophagy or mitochondrial unfolded protein response (mtUPR), in addition to mitochondrial biogenesis that promotes the growth and formation of new mitochondria. Moreover, other renewal mechanisms have recently emerged, such as mitochondrial-derived vesicles (MDVs). The ability of mitochondria to release their contents into vesicles is a conserved process shared with their bacterial ancestors [22]. When mitochondrial stressors are present, mitochondrial inner and outer membranes become oxidized, leading to their loading into vesicles which are transported to lysosomes or peroxisomes for degradation, removing damaged proteins and thus preventing mitochondrial dysfunction [23].

All these processes form part of the protein quality control system of the mitochondrion that is vital for mitochondrial function and cell homeostasis [24]. In this review, we focus specifically on mitochondrial biogenesis and the mtUPR and, in particular, on the implication of mtUPR modulation as a potential treatment of primary and secondary mitochondrial diseases. In addition, we discuss the negative consequences of its activation in cancer patients and its overaction in pathological situations.

## 2. Mitochondrial Proteostasis

### 2.1. Mitochondrial Biogenesis

Mitochondrial biogenesis is defined as the process by which new mitochondria are formed by the growth and division of pre-existing ones, leading to an increase in mitochondrial mass. This process involves the synthesis of new mtDNA and nuclear and mitochondrial-encoded proteins and membranes [25], a complex mechanism that requires communication and coordination between mtDNA and nDNA.

**Figure 1:**
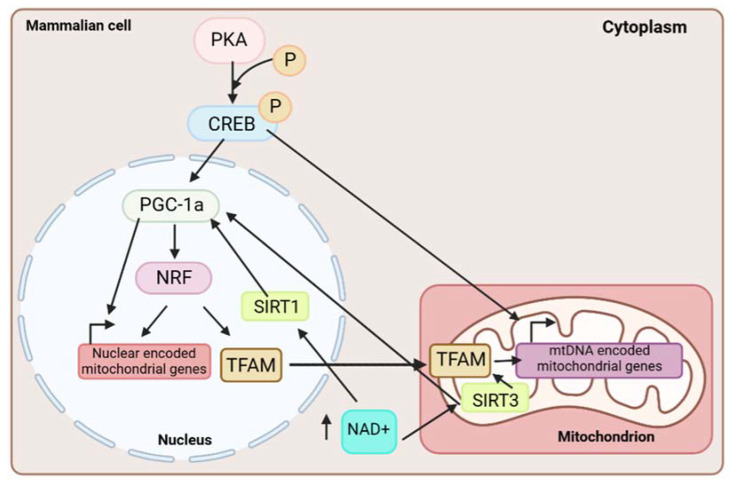
Mitochondrial biogenesis signaling pathway in mammalian cells. In the cytoplasm, PKA phosphorylates and, consequently, activates CREB, which then localizes to the mitochondria and starts the transcription of several mtDNA-encoded mitochondrial genes. Moreover, activated CREB translocates to the nucleus, where it causes the activation of PGC-1α, which, on the one hand, activates the transcription of nuclear-encoded mitochondrial genes and, on the other hand, activates the NRF transcription factor family. NRF factors induce the transcription of nuclear-encoded mitochondrial genes, including TFAM, a factor necessary for the transcription of mtDNA. Finally, in increased NAD+ level conditions, SIRT1/SIRT3 deacetylases are activated and provoke the deacetylation and activation of PGC-1α and the aforementioned signaling pathway. Moreover, SIRT3 causes the deacetylation of TFAM to promote mitochondrial biogenesis.

In mammals, protein kinase A (PKA) phosphorylates and activates cAMP response element-binding protein CREB, which then localizes to the mitochondria, where it starts the transcription of several genes involved in mitochondrial biogenesis, and to the nucleus, where CREB causes the activation of peroxisome proliferator-activated receptor γ co-activator 1 α (PGC-1α) [26]. In turn, PGC-1α interacts with the nuclear respiratory factor (NRF) transcription factor family, which are the main regulators of mitochondrial biogenesis, mainly NRF1 [27]. NRF transcription factors then promote the transcription of numerous mitochondrial genes, especially those encoding for subunits of the mtETC. In addition, once activated, NRF factors regulate the transcription of many mitochondrial proteins, including mitochondrial transcription factor A (TFAM), which induces the transcription and replication of mtDNA [28,29]. Thus, a signaling cascade is produced, where the activation of CREB by PKA leads to the induction of the transcription factor PGC-1α, which, on the one hand, activates the transcription of nuclear-encoded mitochondrial genes and, on the other hand, activates NRF transcription factors. These, in turn, bind to mitochondrial nuclear-encoded gene promoters, activating their transcription and inducing the activation of TFAM, a factor necessary for the transcription of mtDNA. Moreover, it was reported that the sirtuins family also participates in mitochondrial biogenesis, specifically SIRT1 and SIRT3. SIRT1, under conditions of increased nicotinamide adenine dinucleotide (NAD), is activated and phosphorylated at threonine residue 522. Upon phosphorylation, it promotes mitochondrial biogenesis via the deacetylation of PGC-1α and activation of the signaling pathway previously described [30]. The deacetylation of PGC-1α could also be caused by SIRT3, which controls mitochondrial biogenesis via the AMPK pathway [31]. Likewise, SIRT3 deacetylates TFAM, which also induces mitochondrial biogenesis [32] (Figure 1).

On the other side, the trafficking of nDNA-encoded mitochondrial proteins depends on a mitochondrial localization signal (MLS) located at the N-terminal end, which interacts with the TOM complex for entry into the mitochondria [33]. Subsequently, the proteins are translocated through the TIM complex, requiring the inner membrane potential generated by the mtETC [34]. Then, the proteins are moved to the mitochondrial matrix with the help of the Hsp70 chaperone that interacts with the inner membrane TIM complex, facilitating import and folding [35]. Finally, once in the matrix, mitochondrial processing peptidase (MPP) cleaves the MLS. It is important to consider that for the correct function of the proteins in the mitochondrial matrix, their folding is essential, a process which, in addition to Hsp70, needs the participation of chaperonin Hsp60 [36]. Hsp70 and Hsp60 are located in the mitochondrial matrix, and the latter forms a chaperonin complex with Hsp10 [37].

Stress conditions generated by the disruption of the above processes, as well as by mitochondrial uncoupling or the dissipation of the inner membrane potential, render incorrect mitochondrial biogenesis and protein import, phenomena used by cells as sensors of mitochondrial dysfunction to activate their defense mechanisms [38]. If the stress is severe, the cell activates mitophagy, the process of the complete degradation of the mitochondria through mitochondrial autophagy [39]. However, if the stress is moderate, the cell activates an adaptive response known as the mtUPR, which leads to the activation of genes that promote the recovery of mitochondrial function, metabolic adaptation, and innate immunity, among others [40].

### 2.2. Mitochondrial Unfolded Protein Response (mtUPR)

mtUPR regulates mitochondrial proteostasis and was initially defined as a process consisting of a transcriptional response that increased mitochondrial chaperone expression in response to mitochondrial stress produced by the accumulation of unfolded or misfolded proteins within the mitochondria [41]. However, it was recently suggested that the mtUPR also promotes the activation of other proteins, such as proteases, and is involved in a great variety of physiological and pathological processes such as aging [42], neurodegenerative diseases [43], metabolic disorders [44], cardiovascular pathologies [45], and cancer [46], among others. 

**Figure 2:**
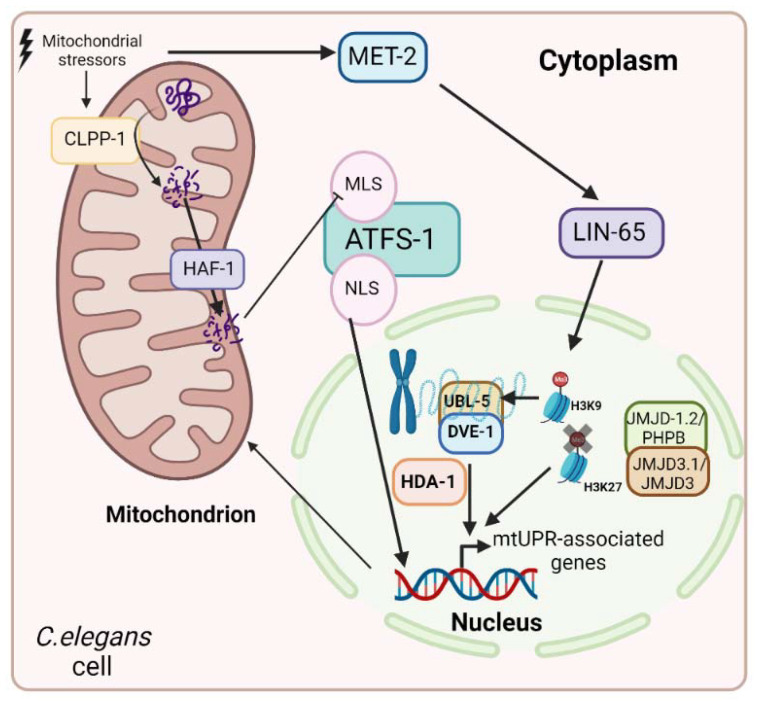
mtUPR in *C. elegans*. When mitochondrial stressors are present, the proteolytic complex CLPP-1 is activated, causing the degradation of proteins to smaller peptides. Then, HAF-1 transports them to the intermembrane space, where the accumulation of small peptides causes the inhibition of ATFS-1 translocation to the mitochondria and its transport to the nucleus, where it activates the transcription of stress response genes. Moreover, there are other factors implicated in mtUPR regulation. Epigenetic modifications, such as methylation and acetylation, are involved in it. In stressful mitochondrial situations, the methylase MET-2 is activated and promotes the translocation of LIN-65 to the nucleus, where it methylates H3K9, promoting chromatin condensation. However, while most of the chromatin is silenced, some parts of it are opened, and it is in those parts where the complex formed by DVE-1 and UBL-5 binds to and activates the transcription of mtUPR-related genes. Otherwise, the lysine demethylases, JMJD-1.2/PHPB and JMJD-3.1/JMJD3, form a complex that demethylases H3K27, promoting the opening of chromatin and the transcription of mtUPR-associated genes. Finally, the histone deacetylase HAD-1 promotes the transcription of mtUPR-related genes via the activation of DVE-1.

The mtUPR pathway was first described in mammals [41,47]. The authors demonstrated the mitochondrial stress-induced activation of mitochondrial, but not cytoplasmic or reticular, chaperone expression. However, the mechanism of the mtUPR was mostly investigated in *C. elegans* [48]. In this organism, different stressors activate the mitochondrial proteolytic complex caseinolytic protease P (CLPP-1), which degrades unfolded and misfolded proteins to smaller peptides of between 8 and 20 amino acids. The matrix peptide ABC exporter hematopoietic-associated factor 1 (HAF-1) transports these peptides from the mitochondrial matrix to the intermembrane space, weakening the import of stress-associated transcription factor 1 (ATFS-1) into the mitochondria [49]. This factor has a nuclear localization signal (NLS) and an MLS. In the absence of mitochondrial perturbation, i.e., under physiological conditions, ATFS-1 is imported into the mitochondria and degraded by the Lon peptidase 1 (LonP1) protease. However, in the presence of stressful mitochondrial situations, the smaller peptides obtained via the action of CLPP-1 reduce the transport of ATFS-1 into the mitochondria, which accumulate in the cytoplasm from where they are imported into the nucleus by its NLS and activates the expression of the stress response [50]. On the other hand, in this pathway, the transcription factor defective proventriculus (DVE-1) is also involved, as well as ubiquitin-like protein 5 (UBL-5). Moreover, epigenetic regulation is implicated in the mtUPR in *C. elegans*. When histone methyltransferase MET-2 or nuclear cofactor LIN-65 are inhibited, the mtUPR is blocked. However, in stressful situations, MET-2 is activated and promotes the entry of LIN-65 into the nucleus and, consequently, the methylation of H3K9. While most of the chromatin is silenced, the remaining parts of it are opened, and it is in these parts that the complex formed by DVE-1 and UBL-5 binds to promote the transcription of mtUPR-related genes [51]. Otherwise, MET-2 is not the unique methylase involved in mtUPR regulation. Two lysine demethylases, JMJD-1.2/PHP8 and JMJD-3.1/JMJD3, are responsible for the demethylation of H3K27, facilitating chromatin opening and the transcription of mtUPR-associated genes [52]. Likewise, the mtUPR is submitted to acetylation regulation. The histone deacetylase HAD-1 exerts its function and promotes the transcription of genes related to the mtUPR in cooperation with DVE-1 [53] (Figure 2).

In mammals, there is an orthologous factor to ATFS-1 called activation transcription factor 5 (ATF5) that has the same function, as well as other transcription factors, such as activation transcription factor 4 (ATF4) and C/EBP homologous protein (CHOP), which regulate the mtUPR by inducing the transcriptional expression of ATF5 [54,55]. However, unlike nematodes, in mammals, it is not clear whether the LonP1 protease is responsible for ATF5 degradation in the absence of mitochondrial damage. CHOP, ATF4, and ATF5 are required for the induction of the mtUPR during mitochondrial damage, although the relationship between them is unclear [56]. It is known, however, that CHOP and ATF4 do not contain an MLS, suggesting that they are regulated at their expression levels and not by mitochondrial import [57]. Several studies have suggested that the phosphorylation of CHOP and ATF4 is necessary and sufficient to induce ATF5 expression, which, once induced, may be translocated to the nucleus to exert its transcriptional function [58,59].

ATF5, ATF4, and CHOP are also related to the integrated stress response (ISR), a conserved adaptive response located in the endoplasmic reticulum that is regulated, similar to the mtUPR, by a variety of stressors [60]. This stress response pathway involves eukaryotic translation initiation factor 2α (eif2α), whose phosphorylation by four different kinases increases the translation of ATF4, ATF5, and CHOP mRNAs and ultimately also activates the mtUPR pathway, promoting the transcription of mitochondrial chaperones (Hsp60 and Hsp70) and proteases (LonP1 and ClPP) [40]. In addition, there are other factors not described in nematodes that regulate the mtUPR in mammals, such as heat shock transcription factor 1 (HSF1), which binds to the mitochondrial single-stranded DNA binding protein 1 (SSBP1), forming a complex that binds to chaperone genes promoters, increasing their expression and thus the misfolding of mitochondrial proteins [61]. In physiological conditions, SSBP1 is located in the mitochondrial matrix, but in the presence of mitochondrial stressors, it is transported to the cytoplasm by voltage-dependent anion channel 1 (VDAC-1) to form the complex with HSF1 and carries out its function in the mtUPR [62].

On the other side, mitochondrial transfer RNAs are processed by RNase P MRPP1, MRPP2, and MRPP3. During mitochondrial stress, the activation of the mtUPR results in a decrease in MRPP3, which is degraded by the LonP1 protease. This prevents the translation of deleterious mitochondrial proteins and promotes the activation of mitochondrial chaperones [63]. Therefore, the mtUPR involves transcriptional and translational regulation. 

Nevertheless, Münch [64] described another two axes of the mtUPR, apart from the ATF5-ATF4-CHOP axis, named the transcriptional canonical mtUPR. In one of them, the estrogen receptor ERα is implicated. This factor is required when mitochondrial perturbations occur in the intermembrane space (IMS), which was described as the intermembrane space UPR. In the presence of mitochondrial stressors in the IMS, the kinase Akt is activated, which phosphorylates and consequently activates ERα. Once activated, this receptor promotes the transcription of NRF1 and the intermembrane space protease OMI, causing the activation of both mitochondrial biogenesis and the intermembrane space mtUPR. Estrogen receptor α and the canonical transcriptional axes may act in parallel. Moreover, mammalian cells without ERα activate CHOP and Hsp60, similar to mitochondrial matrix stress conditions when mitochondrial stress is produced in the IMS [65].

The other axis described by this author implicates sirtuins. The participation of sirtuins in the regulation of the mtUPR was previously described by other authors [66]. In the mtUPR sirtuin axis, the deacetylation of FOXO3A by SIRT3 provokes its localization to the nucleus, where it activates the transcription of antioxidant enzymes, such as catalase and superoxide dismutase 2 (SOD2) [64], activating an antioxidant stress response complementary to the transcriptional canonical mtUPR. These results were previously described by increasing the levels of NAD+ to activate sirtuins in both mammalian cells [67] and *C. elegans* [68]. To test if the canonical and the SIRT3 axes are independent, SIRT3 was inhibited. However, SIRT3 inhibition did not affect the canonical pathway, demonstrating that the two axes are independent of each other, but activated simultaneously in mitochondrial stress conditions [66]. Although Weng et al. [69] suggested that all sirtuins (SIRT1-SIRT7) participate in the mtUPR, only the role of SIRT3 was primarily studied. Therefore, we may conclude that SIRT3 is the main member of the sirtuins family that is involved in the regulation of the mtUPR, and further research is needed to elucidate if the other sirtuins are implicated in this stress response.

**Figure 3:**
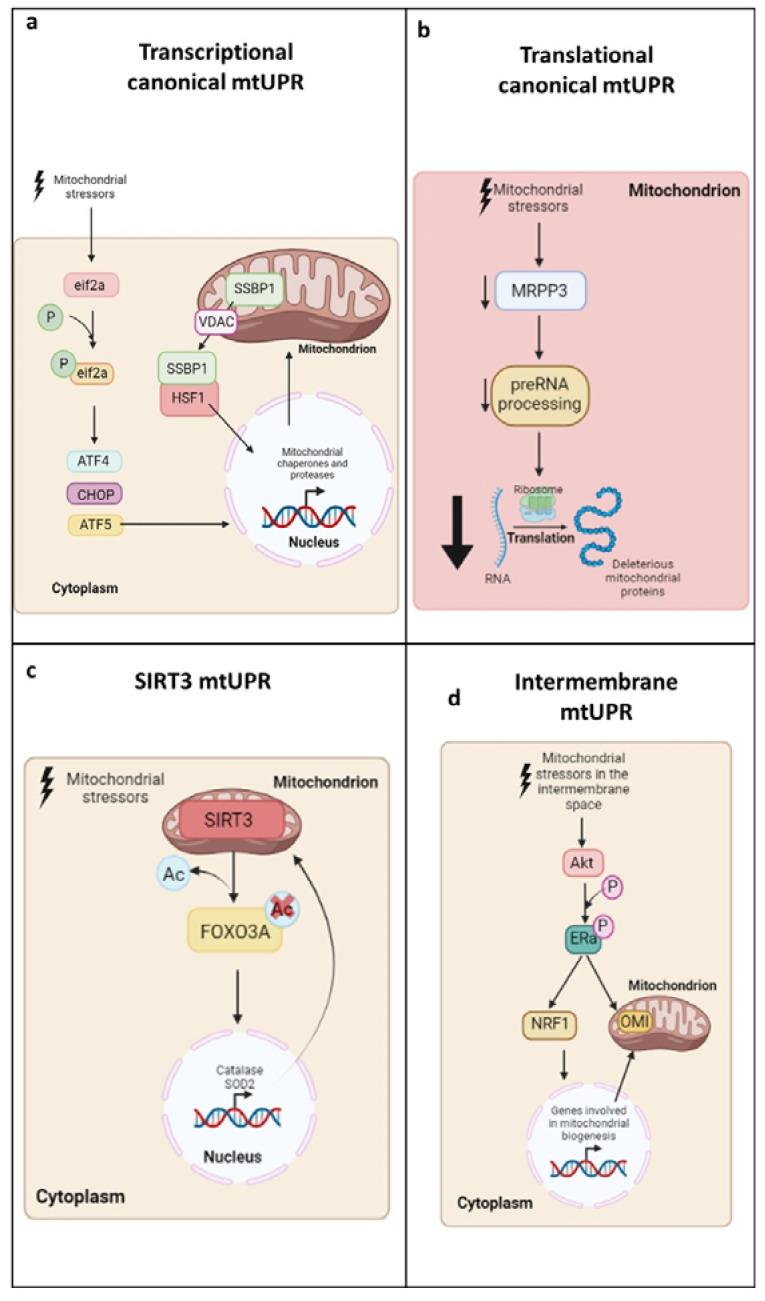
Different axes of the mtUPR in mammals. (**a**) Transcriptional canonical mtUPR. In this pathway, a wide range of stressors provokes the phosphorylation of eif2α, which promotes the activation of ATF4, ATF5, and CHOP. ATF5, thanks to its NLS, is translocated to the nucleus, where it activates the transcription of mitochondrial chaperones and proteases. Moreover, HSF1 also participates in this pathway since it binds to SSBP1, increasing the expression of mitochondrial chaperones. (**b**) Translational canonical mtUPR. In this pathway, the mitochondrial stressors decrease the MRPP3 Rnase, which is degraded by LonP1. As a result, the processing of the mitochondrial preRNA decreases, as well as mitochondrial translation, preventing the presence of deleterious mitochondrial proteins. (**c**) SIRT3 mtUPR. Here, the deacetylation of FOXO3A by SIRT3 results in its transport to the nucleus, where it activates the transcription of antioxidant enzymes, such as catalase and SOD2 (**d**) Intermembrane mtUPR. When the mitochondrial stressors affect the IMS, a different axis is activated. In this, the phosphorylation of ERα by Akt activates NRF1 and the intermembrane protease OMI, promoting mitochondrial biogenesis and the degradation of aberrant proteins, respectively.

In summary, there are different axes of the mtUPR, which could be activated complementarily and in parallel, but are independent of each other (Figure 3).

Furthermore, mitochondrial dysfunction may be transmitted between cells and tissues to induce the mtUPR. This was investigated using neuronal-specific mitochondrial stressors, which provoke intestinal and muscle mtUPR activation through the release of signaling factors [70,71].

The mtUPR is activated by numerous factors that alter mitochondrial proteostasis, mainly the malfunctioning or inhibition of mitochondrial chaperones and proteases [72,73,74]. In addition, other factors are responsible for the induction of the mtUPR, including the overexpression or depletion of ornithine transcarbamylase [75], as well as the overexpression of endonuclease G, which increases the load of damaged proteins [76], which leads to excess ROS [77,78,79] or pathogenic toxins, such as cyanide, produced by *Pseudomonas aeruginosa* [80]. Likewise, the depletion of mitochondrial import systems [81], inhibitors of mitochondrial translation, such as tetracyclines (doxycycline, minocycline, and methacycline), or chloramphenicol [45,82,83,84,85,86,87], inhibitors of the mtETC [71,88,89], the depletion of mtDNA, and the downregulation of ribosomal protein expression also activates the mtUPR pathway [42,79]. On the other hand, itaconate, a metabolite derived from cis-aconitate, induces the mtUPR [90]. In addition, the activation of sirtuins by increased NAD+ levels also promotes the mtUPR, in particular, SIRT1 and SIRT3 activation [66,67,69,91,92,93,94]. Likewise, it was demonstrated that caloric restriction could activate the mtUPR via a miRNA-dependent pathway [95], and the treatment with statins, such as fluvastatin and rosuvastatin, that inhibit the 3-hydroxy-3-methyl-glutaryl-coenzyme A (HMG-CoA) reductase in the mevalonate pathway, activate the mtUPR since they interfere with mitochondrial electron carriers [96]. Other compounds that were reported as mtUPR activators are chlorprothixene, an antagonist of the D2 dopamine receptor, auranofin, an antirheumatic agent [86], as well as auraptene, a natural compound from citrus fruits [97]. Finally, another antioxidant compound that activates the mtUPR is choline, which promotes the SIRT3-AMPK pathway [98] (Table 1).

**Table 1:** mtUPR activators. This table summarizes the different mtUPR activators, including the model organism in which they were experimentally assessed and the mtUPR biological markers that are upregulated in each case.

Activators of mtUPR	Examples	Model Organism	mtUPR Biological Markers	References
Malfunctioning or inhibition of mitochondrial chaperones and proteases		*Drosophila Melanogaster*	Hsp60 Hsp70 ClPP	[72]
	Gamitrinib	Cellular models	Akt LonP1 Hsp60 Hsp70	[73,74]
Overexpression or depletion of ornithine transcarbamylase		Cellular models	ClPP LonP1 CHOP Hsp70	[75]
		*C. elegans*	ATFS-1 Hsp60	[75]
Overexpression of endonuclease G		Cellular models	Akt OMI	[76]
Paraquat		*C. elegans*	ATFS-1 Hsp60 SOD2 CLPP-1 LonP1	[78,79]
		Cellular models	ClPP LonP1 ATF5 Hsp60	[77]
Toxins produced by pathogenic microorganisms	Cyanide	*C. elegans*	ATFS-1 Hsp60	[80]
Depletion of mitochondrial import systems		Cellular models	eif2α ATF5	[81]
Inhibitors of mitochondrial translation	Tetracyclines (doxycycline and minocycline, methacycline), retapamulin, and chloramphenicol	Cellular models	LonP1 ATF5 Hsp60 Hsp70 ClPP LonP1 CHOP eif2α P-eif2α ATF4 SIRT3	[45,82,83,84,85,87]
Inhibitors of the mtETC		Cancer cells	SIRT1 Hsp60	[88,89]
		*C. elegans*	Hsp60 Hsp70 UBL-5 DVE-1	[71]
Depletion of mtDNA or the downregulation of ribosomal protein expression	Ethidium bromide	*C. elegans*	CLPP-1 LonP1 Hsp60	[79]
		*Mus musculus*	Hsp60 Hsp70 UBL-5	[42]
Itaconate		*C. elegans*	ATFS-1 Hsp60 UBL-5 CLPP-1	[90]
Activation of sirtuins	Nicotinamide	Cellular models	SIRT3 OMI FOXO3a SOD2	[66]
		*C. elegans*	SIRT3 FOXO3a SIRT1 SOD2	[68]
		*Mus musculus*	SIRT3 SOD2 SIRT1 FOXO3a Catalase Hsp60 Hsp70 CHOP	[67,92,94,99]
	Pterostilbene	Cellular models	eif2α P- eif2α ATF4 ATF5 CHOP Hsp60 Hsp70 SIRT3 Nrf2	[93]
	ε-viniferin	Cellular models and *Drosophila melanogaster*	SIRT3 SOD2	[95]
Caloric restriction		*Mus musculus*	Hsp60 ClPP	[95]
Inhibitors of HMG-CoA reductase	Fluvastatin and rosuvastatin	*C. elegans*	ATFS-1 DVE-1 Hsp60 Hsp70	[96]
D2 dopamine receptor antagonists	Chlorprothixene	*C. elegans*	ATFS-1 Hsp60	[86]
Antirheumatic agents	Auranofin	*C. elegans*	ATFS-1 Hsp60	[86]
Antioxidants	Auraptene	Cellular models	ATF4 ClPP CHOP	[97]
	Choline	Rat	SIRT3 Hsp60 LonP1	[98]

Consequently, mtUPR activation induces the expression of genes involved in iron-sulfur center biosynthesis, ROS scavenging, and mitochondrial fission, such as the dynamin-related protein (Drp1) and mitochondrial translocase systems, as well as glycolytic enzymes [48]. The induction of glycolytic genes may be related to the activation of an alternative ATP synthesis pathway that facilitates the recovery of mitochondrial function. In addition, the canonical mtUPR reduces the biogenesis of oxidative phosphorylation and Krebs cycle complexes to coordinate the rates of biogenesis and the assembly of the mtETC, promoting the further enhancement of mitochondrial recovery. This is achieved through ATFS-1 in the case of *C. elegans* or ATF5 in mammals, as these transcription factors would bind to the nuclear gene promoters of oxidative phosphorylation and Krebs cycle genes, inhibiting their transcription, although, in mammals, this phenomenon has not yet been demonstrated [100].

**Figure 4:**
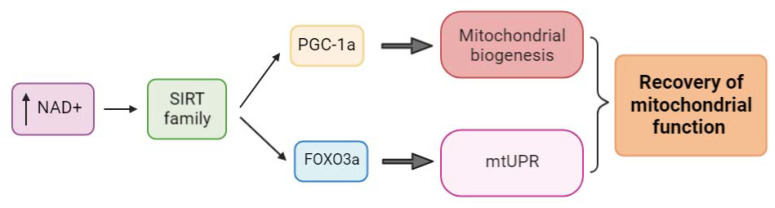
Sirtuins link between mitochondrial biogenesis and the mtUPR. The SIRT deacetylase family is activated by increased NAD+ levels and induces, on the one hand, mitochondrial biogenesis via the deacetylation of PGC-1α caused by SIRT1/SIRT3, and, on the other hand, the mtUPR through FOXO3a deacetylation by SIRT3, improving mitochondrial dysfunction and recovering mitochondrial fitness.

However, on the sirtuin axis, the situation is not the same. In this case, the activation of sirtuins by increased NAD+ levels promotes mitochondrial biogenesis via the deacetylation of PGC-1α and the mtUPR by the deacetylation of FOXO3a, with both pathways acting simultaneously to enhance and recover mitochondrial function [66,69,91,98,101,102,103,104] (Figure 4).

## 3. mtUPR in Primary and Secondary Mitochondrial Diseases

In the following sections, we focus on the relationship between the mtUPR and several physiological and pathological processes, as well as the modulation of this stress response as a potential therapeutic target.

### 3.1. mtUPR and Aging

The decline in mitochondrial function is known to be a characteristic factor of aging, which is associated with the accumulation of mutations in mtDNA as well as with reductions in mtETC and ATP production [19]. This age-related mitochondrial dysfunction may be responsible for the loss of muscle and neuronal function; however, numerous studies have suggested that moderate mitochondrial stress associated with mtUPR activation could delay aging and lead to increased longevity in several biological models. Because aging cells generally accumulate a large amount of unfolded and damaged proteins, it is plausible that the mtUPR is intimately related to aging and age-related diseases [105].

Specifically, in *C. elegans*, it was observed that silencing the complex IV of the mtETC using RNA interference (RNAi) activates the mtUPR and extends its lifespan by approximately 50% [71]. Moreover, the mutation of *ATFS-1* in *C. elegans* shortened the lifespan [106]. The modulation of NAD+ cofactor levels in worms was also shown to activate the mtUPR and extend the lifespan via the activation of *sir2.1*, the homolog of mammalian *SIRT1*, and *daf-16*, the homolog of *FOXO3A* [68]. In mice, damage to mitochondrial ribosomes genetically or pharmacologically and, consequently, the activation of the mtUPR also promoted longevity [42]. Wang et al. [90] suggested that mtUPR activation by itaconate increased healthy longevity in *C. elegans,* improving resistance to different types of stresses and worm motility. Likewise, cultured fibroblasts from the Snell dwarf model showed increased expression levels of Hsp60 and LonP1 [107]. Another example is a study in flies where the disruption of mitochondrial functions and the activation of the mtUPR promoted longevity [108]. 

On the other hand, it was proposed that the mtUPR has a protective role against osteoarthritis (OA). OA is an age-related disease whose prevalence is increasing worldwide, and, unfortunately, there is no effective treatment for it. Mitochondria play a key role in the pathogenesis of this disease since they are crucial for chondrocyte bioenergetics [109]. Zhou et al. [94] showed that the activation of the mtUPR with nicotinamide-protected mitochondria in an OA mouse model stressed chondrocytes as they presented correct structures, such as well-preserved cristae and double membranes, and mitochondrial respiration was restored and mitochondrial membrane potential also improved. In addition, they demonstrated that mtUPR activation alleviates OA pain because it reduces cartilage degeneration and improves chondrocyte survival. Moreover, cartilage tissue from OA patients showed mtUPR activation, which was associated with lower levels of inflammation or reduced chondrocyte death. 

Therefore, there is sufficient evidence that the activation of the mtUPR could be beneficial in slowing aging and extending lifespans in different model organisms, as well as in the treatment of age-related diseases, such as osteoarthritis, although further studies are necessary.

### 3.2. mtUPR and Neurodegenerative Diseases

Given the relationship between aging and the mtUPR, the latter was proposed as a therapeutic option in the treatment of neurodegenerative diseases. Neurodegenerative diseases are characterized by the progressive loss of structures and functions of the nervous system, and a common feature of all of them is the appearance of unfolded proteins and their accumulation in different regions of the brain, depending on the pathology [110]. Furthermore, it was described that mitochondrial dysfunction occurs in neurodegenerative diseases [111]. In fact, it was shown that neurodegenerative disorders are associated with a reduction in the activity of mitochondrial respiratory complexes. In Parkinson’s disease and Alzheimer’s disease, a reduction in the activity of complex I and complex IV was observed, respectively [112]. Furthermore, in Huntington’s disease, the decreased activity of complexes II and III were described [113]. Likewise, the reduction in the activity of α-ketoglutarate dehydrogenase was described in Alzheimer’s and Parkinson’s diseases [114]. Moreover, the pathogenesis of several neurodegenerative diseases was associated with an impaired balance between the mitochondrial fusion and fission processes which promote mitochondrial fragmentation as well as the downregulation of mitochondrial renewal by mitophagy [115]. Therefore, we could consider neurodegenerative diseases as secondary mitochondrial diseases since many lines of evidence suggest that mitochondrial dysfunctions play a key role in their pathomechanisms.

Recent studies suggested that the mtUPR is activated in the neurons of Alzheimer’s disease (AD) patients. In fact, disturbances in the mitochondria, such as decreased mitochondrial respiration, aberrant mitochondrial morphology, or decreased import systems, are known alterations in AD. In the study by Sorrentino et al. [87], they observed that genes related to the mtUPR were upregulated in Alzheimer’s disease, which may be related to a protective response during disease progression. Indeed, ATFS-1 depletion in a *C. elegans* Alzheimer’s model led to a worsening of the disease signs. Furthermore, treatment with doxycycline for the activation of the mtUPR resulted in an increased clearance of Aβ aggregates and increased worm motility [87]. On the other hand, Perez et al. [116] obtained neurons from induced pluripotent stem cells knocked out with the *PITRM1* gene, which codes for pitrilysin metallopeptidase 1. A mutation in this protein generated an age-dependent progressive neurological syndrome, as this protein in humans is involved in the degradation of amyloid-β peptides, leading to an Alzheimer’s disease phenotype. In this study, neurons mutant for *PITRM1* induced mtUPR genes that could act as a protective mechanism for Alzheimer’s disease, as the inhibition of this pathway with ISRIB, an inhibitor of the eif2α kinase, resulted in an increase in Aβ accumulation. Furthermore, treatment of these cells with NAD+ to activate SIRT3 mtUPR enhanced mitochondrial recovery and significantly decreased Aβ and phospho-tau aggregates. Otherwise, treatment with NAD+ or olaparib, an inhibitor of the PARP enzyme, decreased amyloid formation, mitochondrial dysfunction, and aging features in several models [99]. However, Counts et al. [117] suggested that the constitutive activation of the mtUPR could lead to neuronal cell death during the early stages of Alzheimer’s disease. Likewise, Martínez et al. [118] demonstrated that the sustained induction of the mtUPR by introducing an ATFS-1 transcript with no mitochondrial localization signal in a *C. elegans* Parkinson’s disease (PD) model resulted in the death of dopaminergic neurons in a non-caspase-mediated way. This result could be due to the hormetic role of the mtUPR, which could be beneficial in a specific context, but detrimental during its chronic activation. Indeed, the aberrant or prolonged activation of the mtUPR was described as a mechanism of constant mitochondrial recovery that causes the accumulation of deleterious mitochondrial genomes with point loss-of-function mutations or deletions, which is why the precise regulation of the mtUPR is necessary [119]. Nevertheless, in the study by Di Hu et al. [77], the authors observed that the activation of the mtUPR via the deletion of ornithine transcarbamylase in SH-SY5Y cells protected against mitochondrial damage and toxicity produced by MPP+ treatment. This MPP+ treatment initially also induced the mtUPR, but when it was sustained over time, it resulted in increased ROS production and mitochondrial dysfunction, which was mitigated by the overexpression of a vector with the ornithine transcarbamylase deletion. Another example that demonstrates the beneficial role of mtUPR activation in neurodegenerative diseases was provided by Liu et al. [120] in a model of Parkinson’s disease in *Drosophila Melanogaster*. In this PTEN-induced kinase 1 (*PINK1*) mutant model organism, the treatment with ginseng, a Chinese herbal medicine, resulted in an increased lifespan, the rescue of dopaminergic neuron loss, a significant increase of dopamine in the brain, and a delayed onset of the Parkinson’s phenotype. In one mouse model of α-synuclein A53T PD, the overexpression of ClPP ameliorated pathological symptoms via mtUPR activation [121].

Another of the best-known neurodegenerative diseases is Huntington’s disease (HD), caused by the presence (in the majority of cases corresponding to full penetrance) of more than 39 CAG trinucleotide repeats (coding for glutamine) in exon 1 of the huntingtin gene. As a result, an aberrant protein is generated, which provokes its accumulation and leads to DNA damage and mitochondrial dysfunction [122]. There is evidence that the mtUPR is related to the pathophysiology of HD. Thus, Fu et al. [123] observed a downregulation of the mtUPR associated with ROS overproduction and cell death in the striatal cells of a Huntington’s disease mouse model, as well as in the fibroblasts derived from HD patients [124]. In human primary fibroblasts, the expression of the polyQ40 huntingtin gene caused the activation of the mtUPR, demonstrated by the increased expression of mitochondrial chaperones [106]. Naia et al. [103] proposed that SIRT3 activity was increased in HD models, and the translocation of this enzyme to the mitochondrion was also increased in mouse and human cell models. However, in postmortem HD tissues and late-symptomatic mice, there were no changes in SIRT3 activity in comparison with the controls, suggesting that increased SIRT3 activity could be an early adaptative mechanism of the disease. Moreover, the activation of SIRT3 with ε-viniferin improved mitochondrial elongation and anterograde transport in HD striatal neurons. These results were also confirmed in a fly model, demonstrating that SIRT3 and the activation of the mtUPR conferred neuroprotection in Huntington’s disease [103].

Mitochondrial dysfunction was reported in amyotrophic lateral sclerosis (ALS). This disease is characterized by motor neuron death, and most cases are sporadic. However, 5–10% of cases are familial, and up to 20% are caused by mutations in the copper-zinc superoxide dismutase (*SOD1*) gene. The mutation in *SOD1* causes a disruption in the mitochondrial axonal transport in the neuron, leading to an alteration in mitochondrial function and dynamics. Mutant SOD1 is localized in several cell compartments, including the mitochondrion, both in the mitochondrial outer membrane, where it interacts with other proteins, such as VDAC and Bcl2, and in the mitochondrial intermembrane space, where it interrupts the correct folding of crucial mitochondrial proteins [125]. In the study by Riar et al. [126], a G93A-*SOD1* transgenic mouse was used as a model of ALS. In this experimental condition, the activation of the canonical mtUPR occurred as the protein levels of CHOP were greater in the mutant mice than in the control ones. In addition, they demonstrated that mutant SOD1, which accumulated in the IMS, provoked the activation of the ERα mtUPR axis since NRF1 and OMI increased their levels in mutant mice in comparison to the controls. This could be due to the protective mechanism that the cells activate to defend themselves against mitochondrial damage. Zhou et al. [92] proposed that the treatment of the mutant mice with nicotinamide riboside improved neurogenesis, promoted the clearance of the SOD1 mutant protein, and enhanced the mitochondrial function via the activation of the canonical mtUPR. Straub et al. [127] studied patient-derived fibroblasts with mutations in coiled-helix coiled-helix domain-containing protein 10 (CHCHD10), one protein localized in the mitochondrial intermembrane space whose mutation was recently identified as a genetic cause of familial and sporadic ALS. When they cultured these cells in a stress galactose medium, canonical and SIRT3 mtUPRs were activated, probably due to mitochondrial stress. However, the overactivation of the mtUPR by LonP1 downregulation caused the progression of the disease in ALS models [128].

Therefore, all these findings suggest that mtUPR decline is related to the development of neurodegenerative diseases, and its activation could be a potential therapeutic target. However, it was reported that its overactivation might produce several detrimental effects, such as dopaminergic neuronal death in Parkinson´s disease animal models [118], the worsening of disease symptoms in an ALS mouse model [128], or neuronal cell death in AD [117], among others.

### 3.3. mtUPR and Cardiovascular Diseases

Mitochondria play a key role in all tissues but especially in those that have higher requirements of energy, such as the myocardium. Cardiovascular diseases (CVDs) have been associated with mitochondrial oxidative phosphorylation defects [129]. In CVD, mitochondrial dysfunction leads to changes in mitochondrial structure, among them, the formation of megamitochondria due to the overactivation of fusion proteins or enlargement of individuals in restrictive cardiomyopathy or mitochondrial fragmentation and apoptosis in ischemic heart failure [130]. On the other hand, the uncoupling of the mtETC leads to ROS overproduction which promotes atherogenesis by promoting endothelial dysfunction, vessel inflammation, and the accumulation of low-density lipoproteins [131]. 

Since functional mitochondria are essential for cardiac health, the impairment of the mtETC and, consequently, ROS overproduction might be considered key features that result in cardiomyocyte death through apoptosis or necrosis. The mechanisms linking CVD and mitochondrial dysfunction are not entirely clear. However, it was suggested that the reduction in energy supply to the myocardium due to pathological alterations of the mitochondria is responsible for the failure of cardiac function [132,133,134]. For this reason, these pathologies could be considered secondary mitochondrial diseases. 

The relationship between the mtUPR and cardiac disease is sustained since several genes related to the mtUPR were upregulated in both animals and humans with heart pathologies [135]. Likewise, the pharmacological treatment with mtUPR activators, such as choline, that induced the SIRT3 mtUPR axis promoted cardiomyocyte vitality and improved mitochondrial function in animal models of CVD [98]. In one mice model, the activation of the mtUPR with oligomycin or doxycycline alleviated ischemic injury, and this improvement did not occur in the mouse knockout for ATF5, suggesting that ATF5 is a factor necessary for the improvement of the pathophysiology via mtUPR activation [45]. Smyrnias et al. [135] showed that mtUPR activation with small-molecule agents alleviated mitochondrial dysfunction and contractile capacity in murine hearts and demonstrated that in patients with aortic stenosis, reduced plasma biomarkers of cardiac damage, such as levels of abnormal fibrosis or cardiomyocyte cell death, were presented in association with elevated levels of mtUPR-related genes. Therefore, mtUPR activation could also be a potential therapeutic target for the treatment of cardiovascular diseases. However, as well as in neurodegenerative diseases, it was reported that mtUPR overactivation promoted heart failure and cardiomyocyte apoptosis under hypoxic conditions [136,137,138].

### 3.4. mtUPR and Primary Mitochondrial Disease

Primary mitochondrial diseases, caused by mutations in both nDNA and mtDNA, are characterized by mitochondrial dysfunction with a consequent deficiency in ATP production and ROS overproduction [139]. Currently, a prevalence of 1:5000 is established for this type of disease. Unfortunately, probably due to the extreme variety of genes and proteins affected, most primary mitochondrial diseases still lack standard and effective treatments [140]. Moreover, the diagnosis of mitochondrial diseases is challenging due to their clinical heterogeneity and the existence of two genomes implicated in their pathogenesis. 

Nargund et al. [100] showed that in an ATFS-1 mutant worm, mitochondrial genome transcripts increased in response to mitochondrial stress; however, in the wild-type worm, these transcripts only increased modestly, proposing that ATFS-1 is a negative regulator of mitochondrial genome transcript accumulation. Moreover, this factor was also involved in the correct assembly of mtETC complexes during mitochondrial stress by inducing the expression of mitochondrial molecular chaperones. ATFS-1 and, therefore, the mtUPR are essential for the proper transcription of mtDNA and the correct assembly of the complexes encoded by this genome during stressful situations in the mitochondria. Moreover, Suarez Rivero et al. [84], in line with previously published studies by Perry et al. [83], demonstrated that mtUPR activation via treatment with tetracyclines and broad-spectrum antibiotics, improved the pathophysiology of mitochondrial diseases. Specifically, in the study by Suarez Rivero et al., it was observed that treatment with tetracyclines in cell models of the G elongation factor mitochondrial 1 (*GFM1*) mutation increased mtUPR-associated proteins and improved cellular physiopathology. Furthermore, in the former study [83], the authors showed that the treatment with the tetracycline family of antibiotics, as well as the anti-parasitic agent pentamidine and the antibiotic retapamulin, all activators of the mtUPR, improved cell survival in MELAS cybrids and improved Leigh syndrome symptomatology in a complex I-deficient mouse model. 

Another study by Suarez Rivero et al. [93] suggested that mtUPR activation with pterostilbene in combination with mitochondrial cofactors improved the mitochondrial pathophysiology of fibroblasts and induced neurons derived from patients with mitochondrial diseases. Pterostilbene was also reported as a survival and protective compound in several animal models [141] due to its antioxidant, anti-inflammatory, and neuroprotective functions. Moreover, Poveda-Huertes et al. [142] proposed that different stages of mtUPR activation stimulate protein import and cardiolipin remodeling, which could act as beneficial mechanisms in mitochondrial diseases. 

### 3.5. mtUPR and Metabolic Diseases

Mitochondria also play key roles in the pathogenesis of many metabolic diseases due to their central role in essential metabolic pathways. In metabolic disorders, mitochondrial dysfunction induces ROS overproduction associated with a reduction in antioxidant capacity, decreased ATP production, and changes in mitochondrial dynamics [143]. One of the most important metabolic disorders is metabolic syndrome, a compilation of metabolic abnormalities, such as hyperglycemia, insulin resistance, abdominal obesity, hypertension, and atherogenic dyslipidemia [144]. These conditions occur together and increase the risk of cardiovascular diseases and type 2 diabetes (T2D). Several studies proposed a link between mitochondrial dysfunction and metabolic syndrome, although the pathological mechanisms are still unclear [145]. What is known, however, is that metabolic syndrome patients present depressed superoxide dismutase activity, increased lipid peroxidation and carbonylated proteins, as well as increased oxidative damage [146].

T2D, one of the comorbidities of metabolic syndrome, is characterized by insulin resistance in the peripheral tissues as well as elevated blood glucose, which in turn inhibits the function of chaperones and proteases, leading to the accumulation of unfolded and misfolded proteins. These conditions, together with oxidative stress in mitochondria, lead to mtUPR activation to correct these defects [147,148]. In addition, the mtUPR was reported to increase glucose metabolism through the induction of glycolytic enzymes [149]. In this regard, Wardelmann et al. [150] suggested that the mtUPR was decreased in mice after a high-fat diet. However, intranasal treatment with insulin activated the mtUPR in the hypothalamus and reduced weight gain. Moreover, Hauffe et al. [151] demonstrated that reducing the Hsp60 gene protected against obesity and insulin resistance in high-fat dieted male mice. Consistent with the latter results, the LonP1 levels were found to be elevated in the visceral adipose tissue cells of obese individuals, and deficiency of this protease potentiated hepatic gluconeogenesis and caused insulin miss signaling [152]. Additionally, mitochondrial chaperones Hsp60 and Hsp70 also affect T2D since it was proposed that Hsp60 prevents the hyperglycemia characteristic of T2D and its deficiency leads to insulin resistance [153]. Likewise, other components of the mtUPR, such as the sirtuins family, have positive effects on insulin sensitivity [108]. Therefore, the mtUPR could be a potential therapeutic target for metabolic diseases, especially type 2 diabetes.

### 3.6. mtUPR and Cancer

Mitochondrial dysfunction is a hallmark of cancer and is associated with the increased invasiveness, metastatic potential, and drug resistance of cancer cells [154,155,156]. 

First, the mtDNA mutation rate is considerably higher than nDNA, mainly due to the proximity of mtDNA to ROS-generating sites in the mitochondrial electron transport chain. Thus, the accumulation of mtDNA mutations was identified in several cancer types and was associated with metastatic progression and chemoresistance [156,157,158,159] as well as with activating proliferative pathways, such as the AMPK [160] or MAPK [161] signaling pathways.

Furthermore, reductions in oxidative phosphorylation efficiency force cells to depend on glycolysis for ATP production even in the presence of oxygen, which is described as the Warburg effect [162]. However, when mitochondrial dysfunction becomes more severe, excessive ROS production may be lethal to tumor cells [163,164]. Moreover, it was described that mutations in the ND1, ND3, ND4, and ND6 genes of mtDNA promote tumorigenesis and metastasis as well as resistance to apoptosis, contributing to tumor progression [165].

In addition, several mutations in nuclear-encoded mitochondrial proteins are associated with cancer, i.e., mutations in isocitrate dehydrogenase promote leukemogenesis and glioma, and mutations in fumarate hydratase and succinate dehydrogenase facilitate tumor growth and progression via the induction of hypoxia-inducible factor 1α (HIF-1α) [166,167]. 

Due to proteotoxic stress produced in cancer cells by ROS and mutations which hamper the correct folding of proteins, the mtUPR is activated. It was demonstrated that ATF5 was upregulated in a wide range of cancers [168], such as lung cancer [169], pancreatic cancer [170], and carcinomas [171], including ovarian cancer [172], rectal cancer [173], leukemia [174], neural tumors [175,176], esophageal cancer [177], or astrocytoma [178]. Furthermore, ATF5 activation produced resistance to radiotherapy [168] and increased the invasiveness of cancer cells [179]. In addition, Hsp60 was also upregulated in several types of cancers [180], including mammary [181] and ovarian [182] carcinoma, prostate cancer [183], glioblastoma [184] and neuroblastoma [185], and colorectal [186], gastric [187] and pancreatic cancer [188] and this phenomenon is associated with reduced patient survival. Moreover, the knockdown of Hsp60 had beneficial effects, as demonstrated in glioblastoma, where a reduction in this heat shock protein reduced protein translation and cell proliferation [184]. In concordance with these results, Hsp70, also called mortalin, had increased expression levels in different types of cancers [189]. Thus, Hsp70 was upregulated in cancers of the liver [190], ovary [191], and thyroid [192], and its overexpression was associated with lymph node metastasis, advanced tumor stage, and decreased survival [193,194]. Like Hsp60, the knockdown of Hsp70 reduced the migration, proliferation, and invasion of cancer [195]. Finally, LonP1 and ClPP proteases were also upregulated in several types of cancers [196,197,198,199,200,201]. Hence, the activation of the mtUPR is a common feature that occurs in most types of cancer (Table 2), and its inhibition could reduce cancer invasion. Moreover, it was demonstrated that the mtUPR SIRT3 axis is necessary for the invasion and metastasis of cancer cells [202]. For that reason, mtUPR inhibition could be considered a potential therapeutic target for cancer, inducing apoptosis and a reduction in tumor progression and metastasis, as well as increasing patients’ survival.

## 4. Conclusions

Mitochondrial biogenesis and the mtUPR are essential for mitochondrial survival and fitness, the main mechanisms of mitochondrial quality control. Moreover, different mtUPR pathways were proposed, among them, the canonical transcriptional pathway, where ATF4, ATF5, and CHOP participate as the main triggers, the mitochondrial intermembrane space UPR, carried out by ERα as the main participant, the sirtuin pathway, mainly involving SIRT3^69^ and, finally, a translational response that would act when the damage is minor and has a smaller effect. These pathways could act in parallel and complementarily but are independent of each other. Moreover, the sirtuins family, specifically SIRT1 and SIRT3, interconnect mitochondrial biogenesis and the mtUPR via the deacetylation of two key transcription factors, PGC-1α and FOXO3a.

The maintenance of mitochondrial quality is essential since mitochondrial dysfunction may be considered the core of a wide range of pathologies, including primary mitochondrial diseases, metabolic disorders, age-related diseases, cardiopathies, and cancer. In addition, it was reported that mtUPR activation could be a potential therapeutic option for these disorders. Thus, mtUPR activation induces an improvement in physiopathological alterations and a delay in the development of the pathology in many disease models. Nonetheless, its overactivation could be detrimental and worsen both the pathology and symptoms of a wide range of diseases; therefore, precise regulation is necessary. 

However, the situation in neoplastic diseases is the opposite. The activation of the mtUPR leads to the progression and invasion of malignant cells. For this reason, the therapeutic strategy in cancer is based on the targeted downregulation of mtUPR signal molecules instead of its activation. This approach aims to retard tumor growth and induce cell apoptosis.

## Figures and Tables

**Table 2:** Summary of mtUPR factor activation in different types of cancer.

mtUPR Factor Activated	Type of Cancer	Reference
ATF5	Lung	[169]
	Pancreatic	[170]
	Carcinoma	[171]
	Ovary	[172]
	Rectal	[173]
	Leukemia	[174]
	Neural tumors	[175,176]
	Esophageal	[177]
	Astrocytoma	[178]
Hsp60	Mammary	[181]
	Ovary	[182]
	Prostate	[183]
	Glioblastoma	[184]
	Neuroblastoma	[185]
	Colorectal	[186]
	Gastric	[187]
	Pancreatic	[188]
Hsp70	Liver	[193]
	Ovary	[191]
	Thyroid	[192]
LonP1	Colon	[199]
ClpP	Mammary	[197]
	Leukemia	[201]

## Data Availability

Not applicable.
